# The gut microbiome contributes to somatic morphine withdrawal behavior and implicates a TLR2 mediated mechanism

**DOI:** 10.1080/19490976.2023.2242610

**Published:** 2023-08-17

**Authors:** Bridget Truitt, Greeshma Venigalla, Praveen Singh, Salma Singh, Junyi Tao, Irina Chupikova, Sabita Roy

**Affiliations:** a Department of Surgery, Miller School of Medicine, University of Miami, Miami, FL, USA; b Neuroscience Graduate Program, Miller School of Medicine, University of Miami, Miami, FL, USA

**Keywords:** Morphine, withdrawal, microbiome, TLR2, behavior

## Abstract

The ongoing opioid epidemic has left millions of people suffering from opioid use disorder due to the over-prescription of highly addictive substances. Chronic opioid exposure leads to dependence, where the absence of the drug results in negative symptoms of withdrawal, often driving patients to continue drug use; however, few therapeutic strategies are currently available to combat the cycle of addiction and the severity of morphine withdrawal. This study investigates the microbiome as a potential therapeutic target for morphine withdrawal, as gut dysbiosis caused by morphine use has been proven to contribute to other aspects of opioid use disorders, such as tolerance. Results show that although the microbiome during morphine withdrawal trends toward recovery from morphine-induced dysbiosis, there continues to be a disruption in the alpha and beta diversity as well as the abundance of gram-positive bacteria that may still contribute to the severity of morphine withdrawal symptoms. Germ-free mice lacking the microbiome did not develop somatic withdrawal symptoms, indicating that the microbiome is necessary for the development of somatic withdrawal behavior. Notably, only TLR2 but not TLR4 whole-body knockout models display less withdrawal severity, implicating that the microbiome, through a gram-positive, TLR2 mediated mechanism, drives opioid-induced somatic withdrawal behavior.

## Introduction

Over-prescription of opioids for pain management has resulted in nearly 3 million people suffering from opioid use disorder (OUD).^1^ OUD occurs when compulsive drug-taking behavior is chronically displayed, despite negative social, professional, or physical consequences.^2^ Although opioids are effective in treating pain, chronic exposure can result in dependence, which requires the presence of a drug for physical homeostasis. Without the drug present, the body undergoes a withdrawal process that has both physical (headaches, muscle cramps, and shakiness) and psychological (irritability, dysphoria, and drug craving) effects, which disrupt daily functioning.^3^ Additionally, the severity of withdrawal often drives drug-seeking behavior in people with opioid use disorders to find relief from these symptoms, often contributing to the continuation of the addiction cycle.^3,4^ As opioid use increases over time, withdrawal symptoms worsen and cause people to be more motivated to seek drugs to avoid the onset of withdrawal, often resulting in an increase in unsafe drug behaviors.^5,6^

Opioid use disorder is a growing problem, and there are few therapeutic strategies available to combat the addiction cycle and prevent recurrent relapse. Withdrawal treatment is often necessary to stabilize patients with OUD, typically with medications such as buprenorphine, methadone, and naltrexone. However, transitioning between drugs can often result in the onset of withdrawal symptoms that complicate the treatment.^7–10^ Some treatment options require patients to be drug-free for 7–10 days prior to receiving medication, and the withdrawal severity in that time gap results in many failures before treatment begins.^9,11,12^ There are additional complexities regarding access to treatment, with many treatment centers not offering withdrawal medications, and many patients not being able to afford long-term care.^13,14^

In addition to opioid use resulting in OUD, chronic use disrupts the gut microbiome. During morphine treatment, beneficial bacteria decrease in abundance, while pathogenic bacteria flourish, producing a dysbiotic state that decreases gut motility and results in gut barrier dysfunction.^15–17^ Increased permeability of the gut barrier during morphine use increases the risk of bacterial translocation and consequential pro-inflammatory signaling throughout the body.^18^ This morphine-induced dysbiosis perpetuates continued drug use by contributing to the development of tolerance, and pre-clinical research shows that preventing drug-induced microbial changes with antibiotics, probiotics, or fecal microbial transplants can reduce the development of morphine tolerance.[NO_PRINTED_FORM] The relationship between the microbiome and morphine tolerance suggests that the gut microbiome may be a therapeutic target for treating the consequences of OUD.

This study investigated the role of the gut microbiome in morphine withdrawal to test the hypothesis that morphine-induced dysbiosis continues during the early stages of morphine withdrawal and contributes to the development of somatic withdrawal symptoms. This study predicts that preventing these microbial changes will alleviate the severity of withdrawal symptoms, providing a new therapeutic target for treating OUD. Eliminating or even minimizing withdrawal severity would reduce one of the driving forces of continued drug use, facilitating the path to further addiction treatment and recovery.

## Results

### Baseline withdrawal behavior

**Figure 1.**
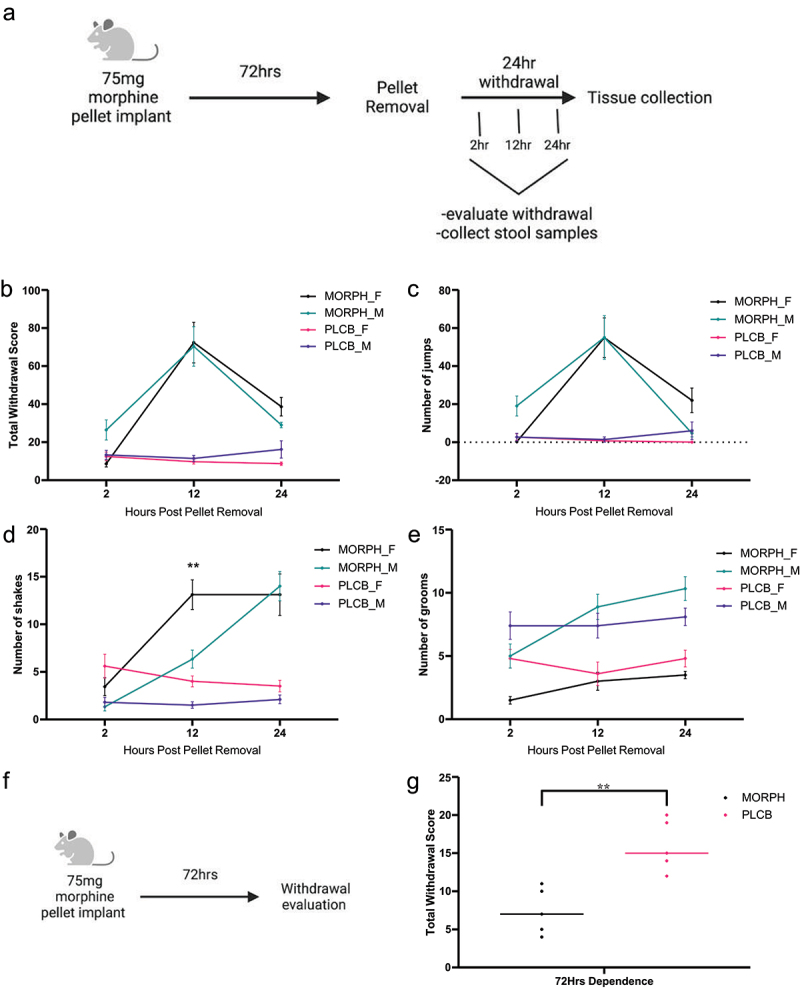
Somatic withdrawal symptoms were observed in female and male mice. Schematic of the withdrawal drug treatment paradigm in four groups: morphine-treated females (MORPH_F), morphine-treated males (MORPH_M), placebo treated females (PLCB_F) and placebo treated males (PLCB_M) (a). Total withdrawal (b), as well as top individual symptoms of withdrawal, jumping (c), shaking (d), and grooming (e). All groups had an *n* = 9–11 animals. Replicates were done with an *n* = 2–3 per group repeated four times. Statistical analysis for all graphs by two-way ANOVA, Tukey’s multiple comparisons test, **p < .01 between the male and female morphine treated animals. Values plotted are mean with SEM error bars. Schematic of the dependence drug treatment paradigm of two groups: morphine (MORPH) and placebo (PLCB) (f). Mean of total withdrawal scores (g). *N* = 5 per group, no replicates. Statistical analysis by students t-test, **p < .01.

To confirm that our morphine treatment paradigm elicited a withdrawal response after pellet removal, somatic symptoms of withdrawal were measured in both female and male mice. Mice were treated with a 75 mg morphine pellet for 72 h, followed by the removal of the pellet and observation of withdrawal symptoms over the course of 24 h (Figure 1a). A two-way ANOVA revealed a significant interaction between the effects of drug treatment and time post pellet removal on the total withdrawal severity [F (6, 102) = 12.86, *p* < .0001]. Multiple comparison results confirmed that morphine treatment resulted in morphine dependence, thus producing a withdrawal response in both male and female mice. Tukey’s multiple comparisons were used to analyze total withdrawal scores (Figure 1b), and the results showed that both male and female mice displayed minimal withdrawal symptoms 2 h post-pellet removal and had no significant difference between sexes (*p* = 0.067) or placebo controls (*p* = .946 for females and *p* = .238 for males). At 12 h post-pellet removal, both male and female mice displayed significantly higher withdrawal symptoms than the placebo-treated controls (*p* < .0001 for females and males), but there was no significant difference between the morphine-treated females and males (0.992). The highest withdrawal scores were observed at 12 h post-pellet removal, and symptoms decreased by 24 h post-pellet removal. At 24 h post-pellet removal, there was no significant difference between male and female morphine-treated mice (*p* = 0.53), but female mice displayed a significantly higher withdrawal score than their placebo controls (*p* = .0002), while the male mice showed no significant difference (*p* = .26).

Individual symptoms of somatic withdrawal were analyzed separately to provide a comprehensive understanding of the somatic withdrawal response at baseline. There were three main symptoms were consistently observed during withdrawal: jumping, shaking, and grooming. Jumping was the most dominant symptom in both the male and female mice (Figure 1c). A two-way ANOVA showed a significant interaction between the effects of drug treatment and time on the total number of jumps [F (6,102) = 10.77, *p* < .0001]. Minimal jumping was observed 2 h after pellet removal, with no significant differences between sexes (*p* = .61) or drug treatments (*p* = .986 in females and 0.116 in males). The most severe jumping behavior was observed 12 h post-pellet removal, similar to the total withdrawal score. There were no significant differences between male and female mice (*p* > .999), and both sexes displayed significantly more jumping than the placebo-treated controls (*p* < .0001 for females and males). Less jumping was observed 24 h after pellet removal, and there were still no significant differences between female and male morphine-treated animals (*p* = .097). Morphine-treated females displayed more jumping than their placebo controls (*p* = .016), and males showed no significant difference between the morphine and placebo groups (*p* = .998). The next most prominent symptom was shaking, although shaking behavior displayed a slightly different pattern compared to the total withdrawal and jumping behavior (Figure 1d). Two-way ANOVA revealed a significant interaction between the effects of drug treatment and timepoint on the total number of shakes [F (6,102) = 13.65, *p* < .0001]. Minimal shaking was observed 2 h post-pellet removal, with no significant differences between male and female mice (*p* = .517) or between their placebo treated controls (*p* = .478 for females and *p* = .99 for males). Shaking behavior increased in both male and female morphine-treated groups 12 h after pellet removal. Morphine-treated female mice displayed a significantly higher shaking behavior than male mice (*p* = .0001). Shaking behavior remained elevated in morphine-treated female mice 24 h after pellet removal and increased in morphine-treated male mice as well, resulting in both morphine-treated groups being significantly higher than their placebo controls (*p* < .0001 in females and males). There was no significant difference between morphine-treated female and male mice at this time point (*p* = .938). The last symptom that was consistently observed was grooming behavior, although morphine-treated mice did not display higher amounts of grooming in male or female mice at any time point (1E). Grooming was consistently observed throughout the withdrawal process, though there was no significant interaction between drug treatment effect and time post pellet removal [F (6,102) = 2.06, *p* = .07]. Excessive grooming can be a symptom of morphine withdrawal, whereas lower levels of grooming are a typical behavior in mice. Although excessive grooming was not observed at baseline, it was measured in future experiments to determine if manipulation of the microbiome caused an increased amount of grooming.

To verify that the withdrawal response after pellet removal was in fact caused by the removal of morphine and could not be observed until after the pellets were removed, withdrawal symptoms were evaluated prior to pellet removal at 72 h of morphine dependence (Figure 1f). Unpaired two-tailed t-tests were used to analyze the withdrawal scores between morphine- and placebo-treated animals (Figure 1g). The results showed that morphine-treated mice not only did not display severe withdrawal symptoms but also showed significantly lower withdrawal scores (*p* = .003). Shaking and grooming behaviors were also lower in morphine-treated animals than in placebo controls (*p* = .011, *p* = .002) (Supplemental 1C, 1D). Since minimal jumping was observed in both groups (Supplemental 1B), the total withdrawal score was mainly influenced by grooming and shaking. There were minimal differences in the withdrawal response in female and male mice; therefore, only male mice were used in the following experiments.

### Microbiome throughout morphine withdrawal

**Figure 2.**
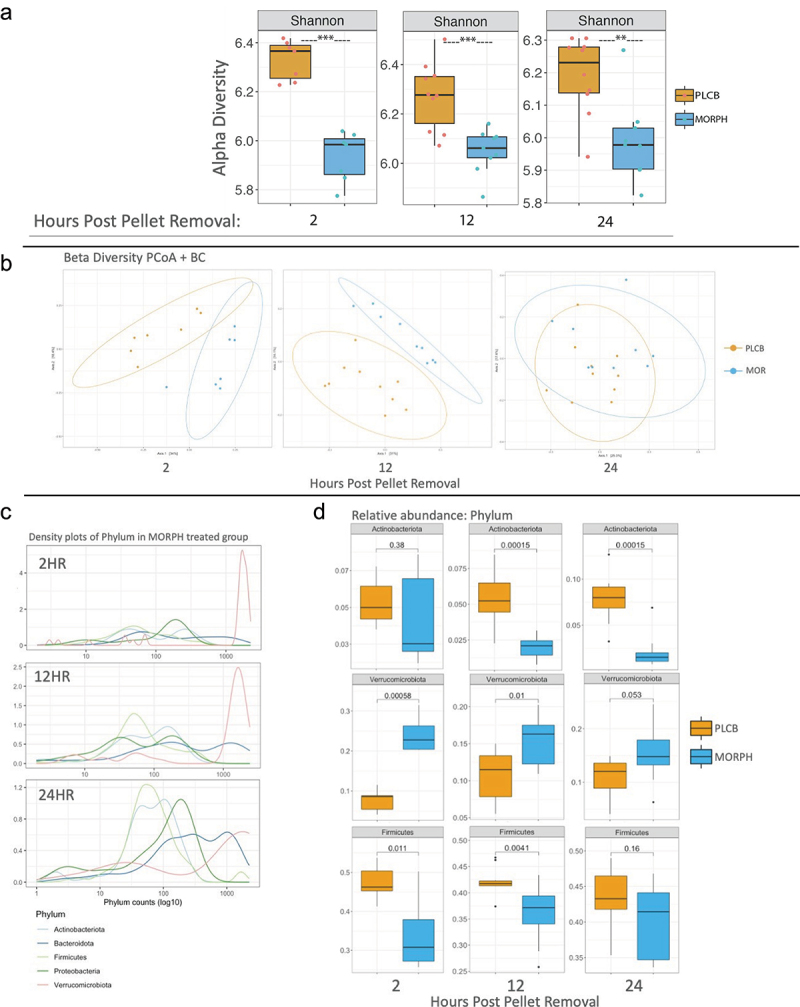
Microbiome shows partial recovery during withdrawal. 16s RNA sequencing of stool samples collected during behavior of two groups: morphine (MORPH) and placebo (PLCB). Box plot of alpha diversity measured by the Shannon alpha diversity index with statistical significance labeled with **p < .01, and ***p < .001(A). PcoA plots of beta diversity using the Bray-Curtis metric (b). All differences shown are significant. The phylum density (c) and box plots of relative abundance (d) with T-test and Mann–Whitney test used for statistical analysis and *p* values numerically represented above graphs. For all graphs in figure: n = 7–10 per group. Replicates were done with an n = 2–3 per group repeated four times.

The composition of the microbiome during morphine withdrawal was analyzed using 16s RNA sequencing. The alpha diversity or species richness in each group was measured using the Shannon alpha diversity index. The results showed that the morphine-treated animals had a gut microbiome environment with significantly lower alpha diversity than placebo-treated animals at 2 h (*p* = 0.0006), 12 h (*p* < .001), and 24 h (*p* = .004) post-pellet removal (Figure 2a). Beta diversity, or the diversity between groups, was analyzed using the Adonis test, and the results indicated that the microbiomes of the morphine and placebo treated animals were significantly distinct from each other at 2 h (*p* < .001), 12 h (*p* < .001), and 24 h (*p* = .022) post-pellet removal (Figure 2b).

Sequencing results at the phylum level provided further details on the changes occurring in the microbiome during morphine withdrawal. The bacterial density in morphine-treated animals showed a shift in prominent bacteria (Figure 2c). At 2 h post-pellet removal, there was an increased density of Verrucomicrobia compared to the other phyla. There was a decrease in the relative density of Verrucomicrobia 12 h after pellet removal, although it remained the most abundant bacteria. By 24 h post-pellet removal, there was a more even destiny of bacteria. The bacterial density of the placebo animals remained unchanged over the 24-h period (Supplemental 1F). Relative abundance analysis of bacteria at the phylum level indicated that morphine-treated animals had significantly higher Verrucomicrobia than placebo treated animals at 2 (*p* = .0006) and 12 h (*p* = .01) after pellet removal. At 24 h post-pellet removal, Verrucomicrobia was still slightly elevated in morphine-treated animals, though not significantly (*p* = .053). Firmicutes, on the other hand, was significantly lower in morphine-treated animals at 2 h (*p* = .011) and 12 h (*p* = .004) after pellet removal, but not significantly different at 24 h (*p* = .16). The abundance of Actinobacteria at 2 h post-pellet removal was not significantly different between morphine- and placebo-treated animals (*p* = .38). At 12 and 24 h post-pellet removals, the morphine-treated animals had significantly decreased actinobacteria (*p* = .0002 and *p* = .0002, respectively). The relative abundances of Proteobacteria and Bacteroidetes were not significantly different between morphine- and placebo-treated animals (Supplemental 1E).

**Figure 3.**
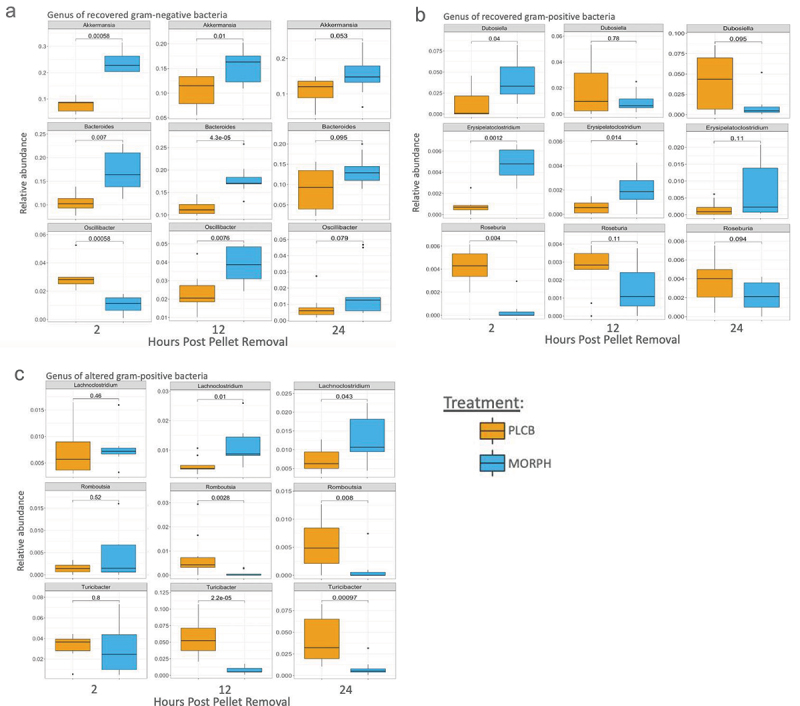
Gram-positive bacteria continue to be altered during morphine withdrawal. The relative abundance of the genus was plotted in box plots and separated into groups, recovered gram-negative bacteria (a), recovered gram-positive bacteria (b), altered gram-positive bacteria (c). T-test and Mann–Whitney test used for statistical analysis with numerical *p* values represented above graph. For all graphs n = 7–10 with four replicates of n = 2–3.

The relative abundance at the genus level was also analyzed between morphine- and placebo-treated animals (Figure 3). One of the trends of abundance observed was that of gram-negative bacterium, Akkermansia, and Bacteroides were significantly elevated in morphine animals at 2 and 12 h after pellet removal (2 h: *p* = .0006, *p* = .007; 12 h: *p* = .01, *p* < .0001) (Figure 3a). Oscillibacter, another gram-negative bacterium, was initially lower in the morphine treated animals at 2 h post pellet removal (*p* = .0006) but became elevated in the morphine group at 12 h (0.008). None of the gram-negative bacteria remained elevated at 24 h after pellet removal (*p* = .053, *p* = .095, *p* = 0.079). A similar pattern was observed for some gram-positive bacteria, including Dubosiella and Erysipelatoclostridium (Figure 3b). Both bacteria were significantly higher in morphine-treated animals 2 h after pellet removal (*p* = .04, *p* = .001). At 12 h post-pellet removal, Dubosiella was no longer significantly different between the groups (*p* = .78), but Erysipelatoclostridium remained significantly elevated in the morphine group (*p* = .014). Neither Dubosiella nor Erysipelatoclostridium was significantly different between morphine and placebo at 24 h post-pellet removal (*p* = .095, *p* = .11). A different trend was observed in the gram-positive bacteria Roseburia, which was significantly lower in morphine-treated animals at 2 h post pellet removal (*p* = 0.004) and was not significantly different at 12 (*p* = .11) and 24 h (*p* = .094). There were also gram-positive bacteria that were not significantly different between morphine and placebo animals at 2 h post-pellet removal, Lachnoclostridium (*p* = .46), Romboutsia (*p* = .52), and Turicibacter (*p* = .8), and became significantly altered throughout withdrawal (Figure 3c). Lachnoclostridium levels increased in morphine-treated animals 12 h (*p* = .01) and 24 h after pellet removal (0.043). Romboutsia and Turicibacter were both significantly decreased in morphine-treated animals at 12 h (*p* = .0003 and *p* < .0001, respectively) and 24 h after pellet removal (p,0.0001, *p* < .001).

### Antibiotics shifted withdrawal timing and duration

**Figure 4.**
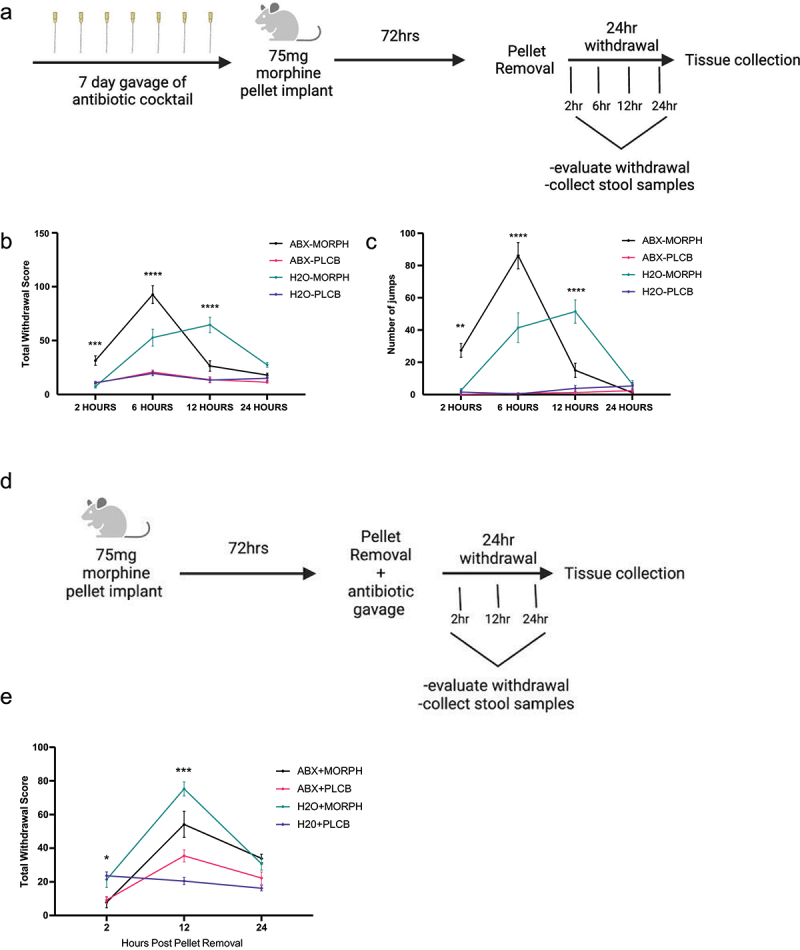
Antibiotics alter the withdrawal response. Schematic of antibiotic and drug treatment for four groups: antibiotic and morphine (ABX+MORPH), antibiotic and placebo (ABX+PLCB), water and morphine (H2O+MORPH) and water and placebo (H2O+PLCB) (a). The mean total withdrawal score (b) and mean number of jumps (c) were plotted with SEM error bars. *N* = 9–10 per group with replicates of 2–3 repeated four times. Statistical analysis by two-way ANOVA, Tukey’s multiple comparisons test, with the significance between the ABX+MORPH and H2O+MORPH groups represented with **p < .01, ***p < .001, ****p < .0001. Alternative antibiotic timepoint with the same four treatment groups: antibiotic and morphine (ABX+MORPH), antibiotic and placebo (ABX+PLCB), water and morphine (H2O+MORPH) and water and placebo (H2O+PLCB) (d). The mean total withdrawal was plotted with SEM error bars. Statistical analysis by two-way ANOVA, Tukey’s multiple comparisons test, *p < .05, and ***p < .001 represents the significant differences between the ABX+MORPH and H2O+MORPH groups. *N* = 9–10 per group with four replicates of 2–3.

To investigate the impact of morphine-induced microbial dysbiosis on somatic withdrawal, mice were treated with an antibiotic cocktail for 7 days prior to morphine exposure to deplete the microbiome and thus prevent microbial dysbiosis caused by morphine treatment (Figure 4a). After antibiotic treatment, the mice were exposed to a 75 mg morphine pellet for 72 h, after which the pellet was removed and somatic withdrawal symptoms were measured 2, 6, 12, and 24 h after pellet removal. Results of a two-way ANOVA showed a significant interaction between the effects of drug treatment and antibiotic treatment on the total withdrawal score [F (9,147) = 21.71, *p* < .0001]. Tukey’s multiple comparison test was used to measure the difference in total withdrawal scores between the groups at each time point after pellet removal (Figure 4b). The results showed that the antibiotic+morphine (ABX+MORPH) group displayed higher withdrawal severity than the antibiotic+placebo (ABX+PLCB) control (*p* = .0027) and water+morphine (H2O+MORPH) groups (*p* = .0002) at 2 h post-pellet removal. H2O+MORPH and its control, water+placebo (H2O+PLCB), were not significantly different at 2 h (*p* = .92). At 6 h post-pellet removal, the ABX+MORPH group still displayed higher withdrawal scores than those of the ABX+PLCB (*p* < .0001) and H2O+MORPH (*p* < .0001) groups. At this time point, the H2O+MORPH group displayed higher withdrawal scores than the control and H2O+PLCB groups (*p* < .0001). At 12 h post-pellet removal, the ABX+MOR group was significantly lower than the H2O+MORPH group (*p* < .0001) but was not significantly different from the ABX+PLCB control group (*p* = .12). The H2O+MORPH-treated group still displayed significantly higher withdrawal scores than the H2O+PLCB control group (*p* < .0001) 12 h after pellet removal. There were no significant differences between any groups 24 h after pellet removal.

The same withdrawal pattern between the ABX+MORPH and H2O+MORPH groups was observed when the jumping behavior was analyzed separately (Figure 4c). Two-way ANOVA results showed a significant interaction between the drug treatment effect and antibiotic effect on the total number of jumps [F (9,146) = 28.82, *p* < .0001]. The ABX+MORPH animals jumped significantly more times than the H2O+MORPH animals at 2 h (*p* = .0001) and 6 h (*p* < .0001) after pellet removal. At 12 h after pellet removal, the ABX+MOR group jumped significantly fewer times than the H2O+MORPH group (*p* < .0001), and there was no difference between these groups at 24 h after pellet removal (*p* = .81). There were also no significant differences in shaking and grooming behavior between these groups at any time point (supplemental 2A-C).

### Antibiotic treatment at time of withdrawal

Since pretreatment with antibiotics prior to morphine dependence is not always a realistic therapeutic option, the impact of antibiotic treatment at the time of morphine withdrawal was investigated to determine if the withdrawal behavior could also be modified via antibiotics after morphine dependence has already developed. To test this, mice were treated with a 75 mg morphine pellet for 72 h, after which the pellet was removed to initiate morphine withdrawal (Figure 4d). At the time of pellet removal, the mice were gavage with 0.2 mL of the antibiotic cocktail, and antibiotics were added to the drinking water to maximize the effects of antibiotics in a short amount of time. There was a significant interaction between the effects of drug treatment and antibiotic treatment on the total withdrawal severity [F (6,98) = 14.29, *p* < .0001]. Tukey’s multiple comparison test of the total withdrawal symptoms (Figure 4e) showed that the ABX+MORPH-treated animals displayed significantly lower withdrawal symptoms than the H2O+MORPH animals at 2 h (*p* = .0464) and 12 h after pellet removal (*p* = .001). There was no significant difference between these groups 24 h after pellet removal (*p* = .92). The ABX+MOR group did not show higher withdrawal scores than the ABX+PLCB group at 2 h (*p* = .993) or 24 h after pellet removal (*p* = .11) but did have significantly higher withdrawal scores at 12 h post pellet removal (*p* = .007). The H2O+MORPH group did not have higher withdrawal scores than the H2O+PLCB control group at 2 h (*p* = .97) but did display a higher withdrawal at both 12 (0.0001) and 24 h post pellet removal (*p* = .023).

When symptoms were analyzed separately, the three main symptoms (jumping, shaking, and grooming) all had a significant interaction between the drug treatment effect and antibiotic effect [F (6,98) = 9.55, *p* < .0001 for jumping], [F (6,98) = 13.03, *p* < .0001 for shaking], [F (6,98) = 6.67, *p* < .0014 for grooming]. Results of the multiple comparison tests showed that the jumping behavior in the ABX+MORPH was lower than the H2O+MORPH, though this was not a significant difference (*p* = .99, 2 h; *p* = .098, 12 h; *p* = .54, 24 h) (supplemental 2F). Shaking behavior displayed a different pattern in which the ABX+MOR group had significantly less shaking than the H2O+MORPH group at 2 h post pellet removal (*p* = .013), no significant difference at 12 h (*p* = .98), and significantly more shaking at 24 h post pellet removal (*p* < .0001) (Supplemental 2 G). Groom behavior showed that the ABX+MORPH group displayed significantly less grooming behavior than the H2O+MORPH group at only 2 h post-pellet removal (*p* = .001) (Supplemental 2 H).

### Microbiome after antibiotic treatment

**Figure 5.**
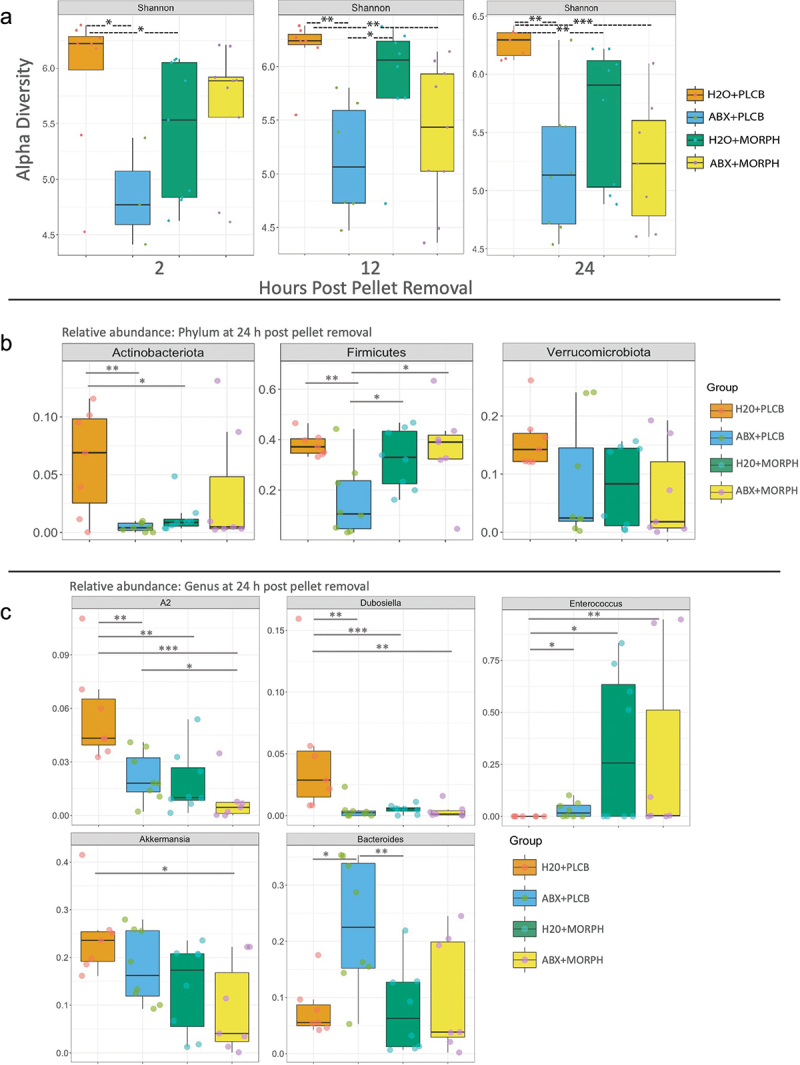
Microbiome after antibiotic treatment. 16s RNA sequencing results of the alpha diversity measured by the Shannon alpha diversity index and plotted in box plots (a). Significance between groups is labeled with *p < .05, **p < .01, ***p < .001. Box plots of the relative abundance of the phylum at 24 h post pellet removal and Wilcox statistical analysis (b). The significant results are indicated by *p < .05, **p < .01, all other differences are not statistically significant. Relative abundance of the genus is displayed in box plot with significant results, of the Wilcox statistical analysis, displayed with *p < .05, **p < .01, ***p < .001, all other differences are *p* > .05. For all graphs in this figure have an *n* = 7–9 with four replicates of *n* = 2–3 per group.

The microbiome was collected from the mice that received 7-day antibiotic treatment prior to morphine exposure and analyzed using 16s RNA sequencing to compare the microbiome changes throughout withdrawal after antibiotic treatment. The Shannon alpha diversity index was used to analyze species richness in each group (Figure 5a). Alpha diversity was consistently lower in the H2O+ MORPH-treated animals than in the H2O+PLCB animals. The H2O+MOPH animals had a significantly lower alpha diversity at 2 h (*p* = .036) and 24 h after pellet removal (*p* = .009). The difference at 12 h post-pellet removal was not significant (*p* = .23), although the mean diversity index was still lower in the H2O+MORPH animals. Additionally, the ABX+PLCB animals had a significantly lower alpha diversity than the H2O+PLCB animals at 2 h (0.048), 12 h (*p* = .005), and 24 h after pellet removal (*p* = .002). The alpha diversity of the ABX+MORPH group was not statistically different from that of the ABX+PLCB and H2O+MORPH groups at any time point, although it was consistently higher than that of the ABX+PLCB group.

The relative abundance was also analyzed to determine the phylum of bacteria remaining in antibiotic-treated animals (Figure 5b). Similar patterns to the microbial changes in the previous microbiome analysis were observed between the H2O+PLCB and H2O+MORPH groups, with decreased actinobacteria after 24 h post pellet removal (*p* = .05) and recovered Firmicutes (*p* = 0.4) and Verrucomicrobia (*p* = .15) after 24 h. As expected, the ABX+PLCB-treated animals showed a lower abundance of most bacteria than the H2O+PLCB animals, having significantly less abundance of Actinobacteria (*p* = .013) and Firmicutes (*p* = .01), as well as lowers average abundance of Verrucomicrobia, though the difference was not significant (*p* = .07). The ABX+MOR group, however, did not show the same antibiotic depletion of bacteria as the ABX+PLCB group and had a microbial composition similar to that of the H2O+MORPH group. The ABX+MORPH group had significantly higher Firmicutes than the ABX+PLCB (*p* = .028) while having similar Firmicutes abundance to the H2O+MORPH group (*p* = 0.78).

The ABX+MORPH group continued to show similar patterns of relative abundance as the H2O+MORPH group at the level of the genus (Figure 5c). Similar to the previous experiment, gram-positive bacteria became altered in the H20+MORPH treated group after 24 h post pellet removal compared to H2O+PLCB (*p* = .001 in A2, *p* = .001 in Dubosiella, and *p* = .05 in Enterococcus). Both the ABX+MORPH and the ABX+PLCB groups also showed lower relative abundance of A2 (*p* = .001 and *p* = .004) and Dubosiella (*p* = .002, and *p* = .004) at 24 h post pellet removal compared to H2O+PLCB. Interestingly, both the ABX+MORPH and the ABX+PLCB groups showed significantly higher abundance of Enterococcus than the H2O+PLCB group (*p* = .01, and 0.02). Gram-negative bacteria were not altered in the H2O+MORPH group compared to H2O+PLCB at 24 h post pellet removal (*p* = .07 in Akkermansia and *p* = .69 in Bacteroides), consistent with the previous experiment. However, the antibiotic treated groups showed different patterns in the gram-negative bacteria, with the ABX+MORPH group displaying significantly lower Akkermansia than the H2O+PLCB group (*p* = .02), though no significant differences compared to the H2O+MORPH (*p* = .54) or the ABX+PLCB group (*p* = .07). Additionally, the ABX+PLCB group had significantly higher abundance of Bacteroides than the H2O+PLCB (*p* = .021) and the H2O+MORPH (*p* = .007) while not being significantly different than the ABX+MORPH group (0.072).

Since then, alterations in the microbiome may have changed the metabolic rate of morphine, causing withdrawal to begin earlier. The plasma levels of the morphine metabolite morphine-3-glucuronide (M3G) were measured using mass spectrometry and analyzed with a t-test. M3G levels were significantly higher in the antibiotic-treated morphine animals than in the water- and morphine-treated controls (*p* = 0.03), indicating a more rapid metabolism of morphine (Supplemental 2D).

### Germ free mice did not display withdrawal symptoms

**Figure 6.**
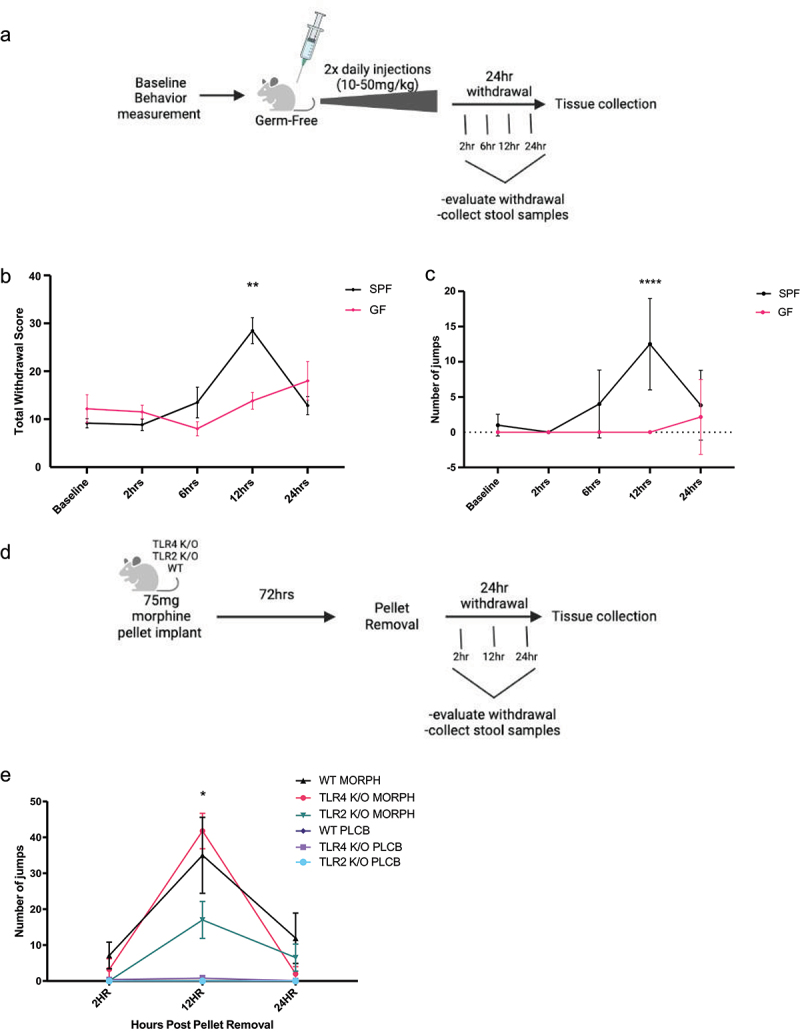
Germ-Free mice and TLR2 whole body knockout mice display less somatic withdrawal symptoms. Germ-Free mouse paradigm with two groups: germ-free (GF) and specific pathogen free (SPF) (a). The mean total withdrawal (b) and mean number of jumps (c) are plotted with SEM error bars and an *n* = 6 per group. Statistical analysis by two-way ANOVA, Tukey’s multiple comparisons test, **p < .01, ****p < .0001 compared between the germ-free and SPF mice. TLR4 and TLR2 whole body knockout model paradigm with six total groups: TLR2 knockout with morphine or placebo (TLR2 K/O MORPH and TLR2K/O PLCB) TLR4 knockout with morphine or placebo (TLR4 K/O MORPH and TLR4 K/O PLCB) and wildtype animals with morphine or placebo (WT MORPH and WT PLCB) (d). Mean number of jumps (e) are plotted it with SEM error bars. Statistical analysis by two-way ANOVA, Tukey’s multiple comparisons test, *p < .05 compared between the TLR2 K/O MORPH and WT MORPH groups. There was an *n* = 9–11 per group. Replicates were run with 1–3 per group repeated five times.

Due to the persistence of bacteria present in antibiotic-treated morphine mice, germ-free mice were used as a microbiome-free model to determine the effects of the lack of a microbiome on somatic withdrawal symptoms. To test this, a baseline measurement of withdrawal prior to morphine exposure was evaluated in both germ-free (GF) and specific pathogen-free (SPF) control mice to serve as a drug control to minimize the number of mice needed for this project, due to the difficulty of breeding and maintaining germ-free animals (Figure 6a). The morphine treatment for the germ-free mice was modified from previous experiments due to high mortality in germ-free mice with the 75 mg morphine pellet model. Instead, mice received a twice-daily IP injection of an escalating dose of morphine, beginning at 10 mg/kg and increasing by 10 mg/kg each day to prevent tolerance. After the last injection of morphine, the mice were evaluated for symptoms of withdrawal 2, 6, 12, and 24 h after the last injection. The total withdrawal score was analyzed between the treatment groups and their baseline scores using a two-way ANOVA and Tukey’s multiple comparison test (Figure 6b). Results of the two-way ANOVA showed a significant interaction between the effect of time and mouse group (SPF or GF) [F(4,50) = 6.11, *p* = .0004]. Multiple comparison test results showed that germ-free animals showed significantly lower withdrawal severity than SPF controls 12 h after the last injection (*p* = .002). The germ-free and SPF mice were not significantly different at the other time points (*p* = .996 at baseline, *p* = .998 at 2 h, *p* = .82 at 6 h, and *p* = .86 at 24 h). Additionally, the germ-free animals did not show higher withdrawal scores than their baseline control at 2 (*p* = .999), 6 (*p* = .96), 12 (*p* > .999), or 24 h after the last injection (*p* = .76), while the SPF animals showed significantly higher withdrawal severity than their baseline score at 12 h after the last injection (*p* < .0001). There was no significant difference between the SPF baseline and other time points after the last injection (*p* > .999 at 2 h, *p* = .95 at 6 h, and *p* = .98 at 24 h). The analysis of jumping behavior alone (Figure 6c) showed the same pattern of withdrawal as germ-free mice jumping significantly less than SPF mice at 12 h after the last injection (*p* < .0001). There was a significant interaction of the effects of time and mouse group (GF or SPF) on jumping behavior [F(4,50) = 6.38, *p* = .0003]. However, the germ-free animals did not jump significantly more times than baseline at any other time point after the last injection (*p* > .999 at 2, 6, and 12 h, *p* = .98 at 24 h), and SPF controls showed significantly more jumping at 12 h after the last injection (*p* < .0001), but not at any other time point (*p* > .999 at 2 h, *p* = .887 at 6 h, and *p* = .917 at 24 h). Shaking and grooming behavior did not show any significant differences between germ-free and SPF animals at any time point after pellet removal (Supplemental 3 A-C).

### TLR2 and TLR4 withdrawal

To investigate the potential mechanisms by which the microbiome modulates morphine withdrawal, the withdrawal response of toll-like receptor 2 (TLR2) and toll-like receptor 4 (TLR4) whole-body knockout models was measured to determine whether gram-negative and/or gram-positive bacteria contribute to morphine withdrawal symptoms. To test this, TLR2 whole-body knockout (TLR2 K/O), TLR4 whole-body knockout (TLR4 K/O), and wildtype (WT) animals were implanted with either a 75 mg morphine pellet (MORPH) or placebo (PLCB) control (Figure 6d). After 72 h, the pellets were removed, and the animals were evaluated for morphine withdrawal at 2, 12, and 24 h after pellet removal. As jumping is the most prominent withdrawal symptom that drives the total withdrawal score, only jumping was scored for these animals. The total number of jumps was analyzed using a two-way ANOVA and Tukey’s multiple comparison test (Figure 6e). The two-way ANOVA revealed a significant interaction between the effects of the genetic strain of mice and the drug treatment on the total number of jumps [F (10, 131) = 5.12, *p* < .0001]. The multiple comparisons results indicated that the TLR2 K/O MORPH animals jumped significantly less than both TLR4 K/O MORPH (*p* = .0007) and WT MORPH animals (*p* = .026). TLR2 K/O MORPH animals also did not jump significantly more than TLR2 K/O PLCB controls at any time point (*p* > .999 at 2 h, *p* = .153 at 12 h, and *p* = 0.971 at 24 h). The TLR4 K/O MORPH animals displayed significantly more jumps at 12 h post-pellet removal than the TLR4 K/O PLCB controls (*p* < .0001) and no significant differences in jumping at 2 h (*p* = .996) and 24 h after pellet removal (*p* > .999). TLR4 K/O MORPH did not have significantly different jumping behavior than WT MORPH animals at any time point (*p* = .985 at 2 h, *p* = .847 at 12 h, and *p* = .521 at 24 h). To verify that the TLR2 K/O mice did not display an earlier withdrawal as seen in the antibiotic-treated animals, the withdrawal behavior was also measured 6 h post-pellet removal in the TLR2 K/O animals (Figure 6f). The TLR2 K/O MORPH animals still showed a lower average number of jumps than the WT MORPH controls, although this difference was not significant (*p* = .231).

## Discussion

### Pellet removal produced morphine withdrawal

In this study, we examined the composition of the microbiome in the first 24 h of withdrawal and investigated the impact of the microbiome on the somatic withdrawal behavior. We confirm that our treatment paradigm results in dependence and withdrawal, which is consistent with other studies that focus on chronic morphine treatment and exposure.^19^ The chronic morphine treatment used in this study (continuous release) is more clinically relevant to chronic pain management than other injection paradigms that replicate recreational use of morphine. Interestingly, withdrawal from slow-release morphine pellet is more severe than an injection paradigm.^20^ Our study did not show significant sex differences in the withdrawal behavior, however it is important to note that the mechanism that drives the behavior may differ between females and males, as other studies have shown sex differences in non-behavioral-based withdrawal measurements.^21,22^ Other contributing factors that can alter behavioral results between the sexes include the animal model used or the treatment paradigms.^21,23–25^

### The microbiome partially recovers during morphine withdrawal

Our results show that morphine-induced dysbiosis partially recovers throughout morphine withdrawal. The decreased alpha diversity and distinct beta diversity reflect the continued disruption of the microbiome following morphine treatment, as both changes are consistently seen in the literature.^15,16,26^ Additionally, gram-negative and gram-positive bacteria were altered at the beginning of morphine withdrawal, which is also consistent with dependence-induced microbiome changes; however, throughout morphine withdrawal, the persistence of pathogenic gram-positive bacteria, suggests a more important role of gram-positive bacterial communities in the withdrawal response. However, it is possible that some bacterial communities can change independently of the behavioral response and not every difference observed in the microbiome during morphine withdrawal can be attributed to the somatic withdrawal symptoms.

### Antibiotic treatment altered the somatic withdrawal response

Antibiotic treatment did result in a change in withdrawal timing and severity, indicating the microbiome is involved in the modulation of somatic withdrawal symptoms. Both pre-treatment of antibiotics and antibiotic treatment at the time of pellet removal resulted in changes to the somatic withdrawal response, showing intervention at the level of the biome can occur before or after dependence on morphine is established. This flexibility of intervention is crucial for the various conditions in which morphine withdrawal can occur. Future research into additional methods of microbiome manipulation, such as probiotics, prebiotics, or fecal microbiota transplant, at different timepoints of morphine treatment and withdrawal would provide a better understanding into what specific bacteria are contributing to morphine withdrawal and the ideal treatment timing and method.

The composition of the microbiome after antibiotic treatment differed slightly between the placebo and morphine treated groups, showing that the microbiome in the antibiotic treatment did not have as successful of a depletion of the microbiome under morphine treatment compared to placebo. It is possible that the remaining bacteria in the antibiotic and morphine treated group could explain why there is remaining withdrawal behavior observed in these mice, so germ-free mice were used in the following experiment to address this possibility. Interestingly, both morphine treatment and antibiotic treatment alone cause decreases in the alpha diversity, however the combination of morphine and antibiotics did not result in the lowest alpha diversity. Since morphine treatment also causes increases in pathogenic bacteria,^15,16^ it is likely that the antibiotic treatment limited the abundance of commensal bacteria, allowing for more species of pathogenic bacteria to thrive and increase the overall species richness. The lack of competition from the commensal bacteria also explains why the antibiotic plus morphine treated group had higher relative abundance of some phyla and genus. The morphine and water treatment groups showed similar patterns in alpha diversity and altered gram-positive bacteria as was shown in the first experiment. Discrepancies in the specific phylum altered between experiments can be caused by different morphine-induced changes in the species within each phylum, or batch differences between cohorts of animals.

### Somatic morphine withdrawal was attenuated in germ-free animals

The antibiotic-treated animals showed that somatic withdrawal could be altered by modifying the microbiome, but the remaining withdrawal symptoms could be explained by the persistent bacteria present. To determine whether the microbiome is required for somatic withdrawal, the withdrawal response was measured in germ-free animals that lack a microbiome from birth and are maintained in a germ-free facility to prevent the development of a microbiome. The somatic withdrawal response was fully attenuated in germ-free animals as they did not display withdrawal scores higher than baseline at any time point of withdrawal. Additionally, they displayed minimal jumping behavior, which is the behavior most prominent during withdrawal and only present in high numbers in morphine-treated animals. This confirmed that the microbiome is necessary for the development of somatic withdrawal symptoms. However, since germ-free animals are germ-free from birth, we cannot test the impact of a germ-free environment on somatic withdrawal after morphine dependence has developed, meaning the lack of somatic withdrawal in the germ-free mice could be due to an inability to develop dependence. Even with the lack of dependence, the conclusion remains that somatic morphine withdrawal does not occur in the absence of a microbiome. Additional limitations of germ-free animals include health defects that result from a lack of a microbiome, as both immune function and brain development both require a homeostatic microbiome to function effectively.^27,28^

### Somatic withdrawal is decreased in TLR2 but not TLR4 knockout animals

We found that a TLR2 whole-body knockout resulted in attenuated withdrawal severity, while the TLR4 knockout did not. This suggests a more important role of the gram-positive bacteria over the gram-negative bacteria, a pattern also seen in a tolerance model by Zhang et al. 2019 in which both TLR2 and TLR4 knockout showed partial attenuation of morphine-induced analgesic tolerance, but the TLR2 knockout mice showed significantly more attenuation. In our study, gram-positive bacteria remain altered throughout morphine withdrawal, and a whole-body knockout of the TLR2 receptor decreases the severity of morphine withdrawal, suggesting a potential mechanism in which the microbiome modulated somatic withdrawal.

There is some variation in the withdrawal severity in the control animals throughout these experiments, which was expected as different cohorts of animals often differ in baseline behavior based on environmental conditions.^29–31^ To best account for baseline differences, we took a number of precautions to ensure these differences would not impact the experimental findings. First, we chose a strain of mice that ranked low on the within-strain variability,^29^ and ensured all mice were habituated to our housing facility for 1 week prior to any experiments. Additionally, for each experiment, we ensured all treatment groups were run with the same experimenter in the same behavioral room and equipment. Groups were run with a small n and multiple replicates, so each treatment condition was reflected in every replicate. There were some exceptions where groups were run separately, but immediately following the first groups, as male and female mice cannot be behaviorally evaluated at the same time, and the germ-free mice and SPF controls needed to be run separately to prevent contamination to the germ-free animals. There can also be some variation in the scoring of the behavioral videos over time and different experimenters, so we ensured the scorer was always blinded to the treatment conditions and was the only scorer for that set of experiments which were scored in a short period. Finally, we had the appropriate control groups for each experiment, so the analysis could be done with controls run in the exact same conditions, and any environmental variation seen in the mice would be reflected in all treatment groups.

In conclusion, this study showed that the microbiome contributes to somatic morphine withdrawal potentially through a gram-positive, TLR2 mediated mechanism. There is a partial recovery of the microbiome during morphine withdrawal and manipulation of the microbiome during morphine treatment causes changes in the withdrawal timing and severity. Future studies will aim to therapeutically treat the microbiome more specifically to further minimize or prevent the development of somatic withdrawal symptoms.

## Methodology

### Animals

Mice used were C57BL/6 wild-type mice purchased from Jackson Laboratory. Both male and female mice were used between the ages of 12-15-week old and were housed in a facility with a 12 h light dark cycle. Mice were given unrestricted access to food and water throughout all experiments and were given fresh bedding once a week or daily during antibiotic treatment.

Germ-free mice were bred and housed in a germ-free facility until the beginning of morphine treatment at 13 weeks old, where they were housed in a specific pathogen-free (SPF) facility. The bedding of the germ-free cages was changed daily, and the cages were opened under a sterilized hood to eliminate germ exposure. The behavioral chambers were sterilized prior to any contact with the mice, and no other animals used the chambers during the germ-free experiments. These mice were given non-restricted access to autoclaved food and water and were maintained on a 12 h light/dark cycle. The mice were group-housed with germ-free mice.

Whole-body TLR4 knockout animals used were the Tlr4^tm1.2Karp^ targeted knockout line purchased from Jackson Laboratory. Genotyping confirmed the deletion of TLR4 (Supplemental Figure 3e). Whole-body TL2 knockout animals were bred in-house, and deletion of TLR2 was verified by genotyping (Supplemental 3F). Both knockout strains were housed in the same facility as wild-type mice, given non-restricted access to food and water on a 12 hr light dark cycle and weekly cage cleaning.

All animals were monitored for general health and signs of distress throughout the withdrawal period, including weight loss, lack of drinking and/or eating, excessive vocalization, and fighting/injury. Only minor weight loss was observed throughout withdrawal, which was expected as this is a documented symptom of withdrawal. At the end of the experiment, the mice were sacrificed using a carbon dioxide chamber, followed by cervical dislocation, and tissue samples were collected.

### Drug treatment

Wild-type mice were treated with a 75 mg slow-release morphine pellet for 72 h to ensure drug dependence. The control mice received placebo pellets which dissolve at the same rate as the morphine pellets while releasing an inactive substance. All pellets were encased in a nylon mesh prior to implantation to facilitate the removal of pellets after 72 h. To implant the pellet, the mice were anesthetized with isoflurane, and a small incision was made on the back of the mice below the neck. Scissors were inserted into the incision and opened gently to create space for the pellet at the nape of the neck. The pellet was inserted into the incision, and the incision was closed using a surgical staple. The mice were removed from anesthesia and returned to their cages for recovery. Heath checks were performed to monitor the well-being of animals over the course of morphine exposure. The pellets were removed after 72 h to begin spontaneous withdrawal. To remove the pellets, mice were anesthetized with isoflurane, and another incision was made at the base of the pellet. Forceps were used to grab and remove the pellet through an incision. Once the pellet was removed, the incision was closed with a surgical staple, and the mice were removed from anesthesia and returned to their cage. The timing of morphine treatment was kept consistent to ensure all behavioral evaluations of withdrawal were done at the same time of day between replicates. Pellets were implanted at 7am and removed at 7am 3 days later.

Germ-free mice did not receive morphine pellets because the dose of the pellets was lethal to mice without a microbiome. Morphine powder was dissolved in saline and injected intraperitoneally. Germ-free and SPF control mice received a twice-daily escalating dose of morphine, beginning at 10 mg/kg and increasing by 10 mg/kg for 5 days, resulting in a final dose of 50 mg/kg. The dose was increased daily to prevent the development of tolerance. Injections were administered every 12 h (7am and 7pm) to minimize withdrawal between doses. Treatment began at 7pm with the first injection, so that the last injection was given at 7am and behavioral evaluation of withdrawal could follow the same timepoints as the pelleted experiments.

### Withdrawal measurement

The somatic signs of morphine withdrawal were measured periodically over a 24 hr period following pellet removal (at 2, 12, and 24 h post pellet removal) or the last morphine injection (2, 6, 12, and 24 h after the last injection). Symptoms of morphine withdrawal were evaluated by placing the mice in a plexiglass cylinder for a 15-min period, behavior was recorded, and mice were returned to their cage. The behavioral videos were then scored by a blinded observer based on Table 1, modified from Taylor et al., 1998.^32^ Symptoms of withdrawal were evaluated based on the frequency of the symptom (counted signs) or the presence of the symptom (checked signs), which resulted in a quantifiable score of withdrawal, with a higher score indicating more severe withdrawal.

**Table 1.** Table of withdrawal symptoms used to score the behavioral videos. This list of symptoms is adapted from Taylor et al. 1998, and it is compiled of two categories of symptoms.^32^ Counted signs are scored based on the frequency of these behaviors and are observed in which each time the symptom is recorded results in a + 1 of the total withdrawal score. Checked signs are evaluated based on the presence of the symptom and a point is added to the withdrawal score if the symptom is seen at all. This results in a numerical total withdrawal score, with a higher score indicating a more severe withdrawal.

**Counted Signs**	
Wet-dog Shakes	Shakes of head or the entire body
Grooming	Using limbs to manipulate head or body
Jumping	Raising all four paws off of the ground rapidly
Dig	Using forepaws to Scratch the floor of the container
Freeze	Immobility for more than 10 seconds
Rubbing	Moving the jaw or the torso on the ground
**Checked Signs**	
Diarrhes	Watery feces
Ptosis	Squinting of the eyes
Irritability	Vocalization when placed in or removed from chamber
Lacrimation	Appearance of brown secretion from eyes
Rhinorrhea	Appearance of brown secretion form nose
Abnormal Posture	Lying on side, writhing,or hunching of body

### Microbiome analysis

Stool samples were collected during behavioral evaluations of withdrawal to analyze the composition of the microbiome throughout the withdrawal process. The stool samples were flash frozen at the time of collection and stored at −80°. Once all stool samples were collected, they were processed for 16sRNA sequencing using the protocol described by Wang et al. (2018).

*Bioinformatics*: Demultiplexed sequence reads were clustered into amplicon sequence variants (ASVs) using the DADA2 package (version 1.26.0) in R (version 4.1.0).^33^ The quality profiles of the forward and reverse reads were visualized using the plotQualityProfile. The steps of the DADA2 pipeline included error filtering, trimming, learning of error rates, denoising, merging of paired reads, and removal of chimeras. The filtering function filterAndTrim was used with the following filtering parameters: maxN = 0, truncQ = 2, rm.phix=TRUE, and maxEE = 2. For paired-end reads, truncLen 200, 190 was used to overlap after truncation to merge forward and reverse reads, respectively. A parametric error model (err) was used to determine the error rate. These error rates were used to infer unique reads from samples. Merging was performed by aligning the denoised forward reads with the reverse complement of the corresponding denoised reverse reads, and then constructing the merged “contig” sequences. Merged data frames construct an amplicon sequence variant (ASV) table, a higher-resolution version of the OTU table produced by traditional methods. Chimeric ASVs were identified and removed. Taxonomy assignment was performed using function assignTaxonomy to the SILVA 138 reference database including species with 99% similarity and for species exact match to improve accuracy.

Statistical analysis was performed using R software (version 4.1.0) with the Phyloseq package 1.38.0. Relative abundance was calculated for ASVs, and normal distribution was tested using the t-test, and those that were not normally distributed were tested using the Mann–Whitney test. The distribution of diversity indices is plotted as box plots. Beta diversity analysis was performed using the Bray-Curtis metric to analyze dissimilarity between the groups. A permutation test (1234 interactions) was used to perform a significance test using the vegan package. All diversity metrics were calculated using the R vegan package, and all plots were generated using the ggplot2 package.

### Antibiotic treatment

To deplete the microbiome to determine the impact on withdrawal severity, mice were treated with an antibiotic cocktail that targets both gram-negative and gram-positive bacteria, as well as an antifungal agent to prevent unwanted fungal growth in the absence of the microbiome. The antibiotic cocktail was prepared using 10 mg/mL bacitracin, 10 mg/mL metronidazole, 40 mg/mL neomycin sulfate, 4 mg/mL Vancomycin, and 24 ug/mL pimaricin dissolved in water. Mice were gavaged with 0.2 mL of the antibiotic cocktail for 7 days prior to morphine treatment, and control animals were gavaged with water. Once morphine treatment began, the antibiotics were maintained in drinking water with 0.5 mg/mL bacitracin, 2 mg/mL Neomycin, 0.2 mg/mL vancomycin, 1.2ug/mL pimaricin added to the water bottles in each cage and replaced daily. The volume of drinking water was measured each day to ensure the consumption of antibiotics and water. During antibiotic treatment, the bedding of the cages was changed daily to eliminate reconstitution of the microbiome via coprophagic behavior.

### Statistical analysis

Prism 9.5.1 was used to perform the statistical analysis of the behavioral data and the correlations. Details on the specific analysis done in each figure can be found in the figure legend. Outliers were not removed from data sets, as both behavioral and microbiome data can have large baseline differences as well as a large range of morphine-induced changes. However, it was ensured that the inclusion of the outliers did not change the statistical significance of the results.

## Supplementary Material

Supplemental Material

Supplemental Material

## Data Availability

Sequence data are reposited at the Biostudies database (https://www.ebi.ac.uk/biostudies/) under accession number S-BSST1068 (https://www.ebi.ac.uk/biostudies/studies/S-BSST1068).
