# Supplementary material for: The gut microbiome contributes to somatic morphine withdrawal behavior and implicates a TLR2 mediated mechanism
Source: Gut Microbes. 2023 Aug 17;15(1):2242610. doi: 10.1080/19490976.2023.2242610 (PMC10438851; doi:10.1080/19490976.2023.2242610)

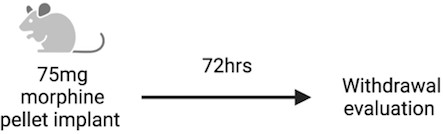
A B ns

**8**

**6**

**Number of jumps**

**4**

**2**

**0**

MORPH PLCB

**-2**

**72Hrs Dependence**

C D

**

*

##### 15

**10**

MORPH

**8** PLCB

**Number of shakes**

**Number of grooms**

##### 10

**6**

#### MORPH PLCB

**4**

##### 5

**2**

**0**

**72Hrs Dependence**

**0**

##### 72Hrs Dependence


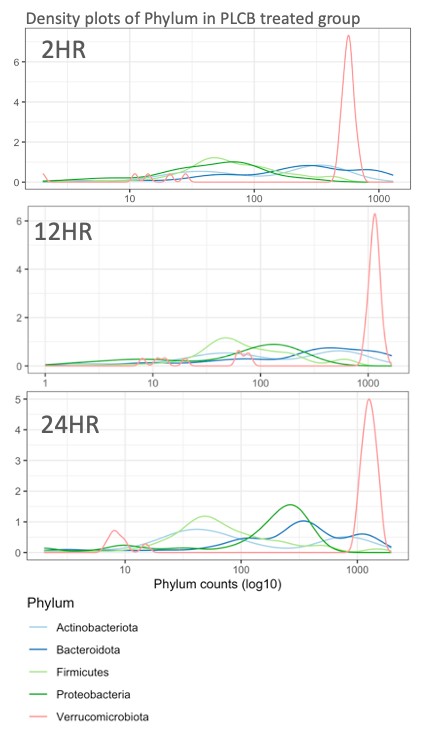


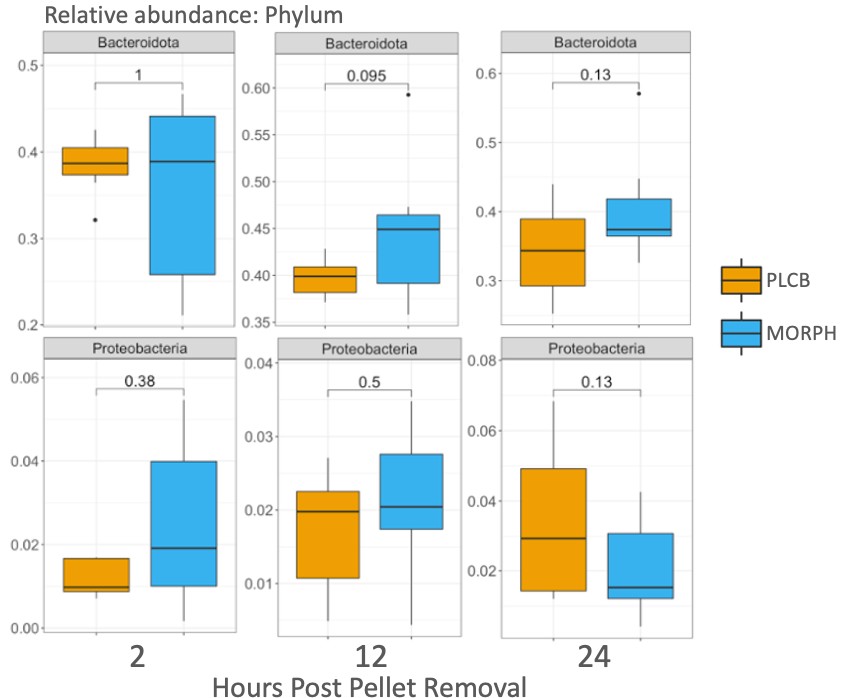


E

F

A B **25**


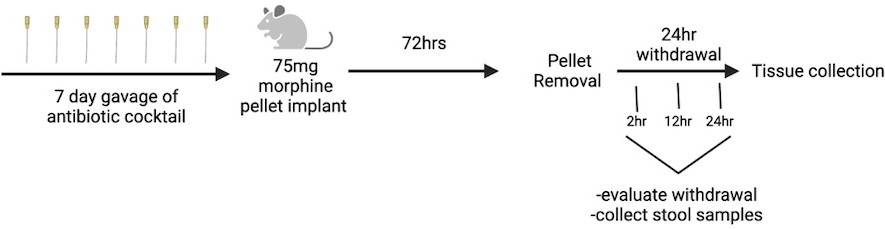
**20**

**Number of shakes**

**15**

#### ABX-MORPH ABX-PLA H2O-MORPH H2O-PLA

**10**

**5**

C **15**

**10**

**Number of grooms**

ABX-MORPH ABX-PLA H2O-MORPH H2O-PLA

**0**

# D

##### 40000

**30000**

**M-3-Glu (nM)**

**2 HOURS 6 HOURS 12 HOURS 24 HOURS**

**Hours Post Pellet Removal**

#### H2O+MORPH ABX+MORPH

##### 20000

**5**

##### 10000

**0**

**2 HOURS 6 HOURS 12 HOURS 24 HOURS**

##### Hours Post Pellet Removal

**0**

##### 2 Hours Post Pellet Removal

E F **60**


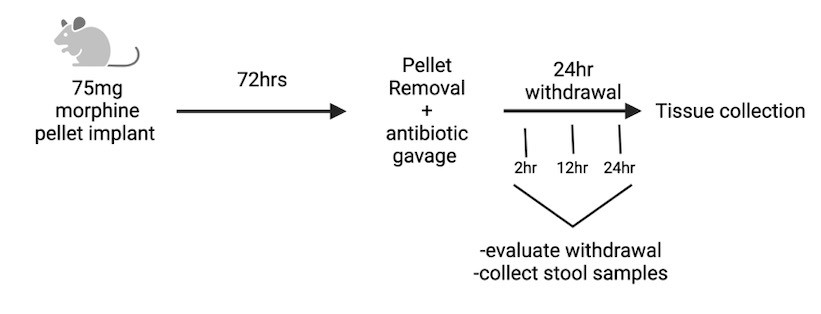
**40**

**Number of Jumps**

**20**

**0**

**2 12 24**

Hours Post Pellet Removal

ABX+MORPH ABX+PLCB H2O+MORPH H20+PLCB

G **30**

*

*

**20**

**Number of Shakes**

**10**

ABX+MORPH ABX+PLCB H2O+MORPH H20+PLCB

**20**

**15** **

**Number of Grooms**

**10**

**5**

ABX+MORPH ABX+PLCB H2O+MORPH H20+PLCB

**0**

**2 12 24**

Hours Post Pellet Removal

**0**

**2 12 24**

Hours Post Pellet Removal

#
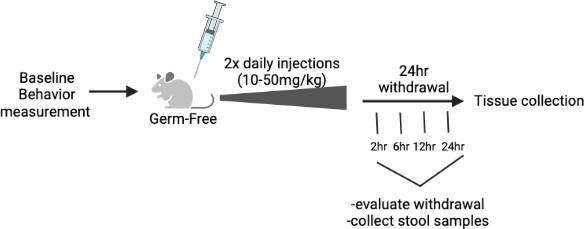
A

B **15**

### 10

**Number of shakes**

C

**15**

GF SPF

**Number of grooms**

**10**

## GF SPF

### 5

**5**

### 0

**Baseline 2hrs 6hrs 12hrs 24hrs**

### 0

**Baseline 2hrs 6hrs 12hrs 24hrs**

#
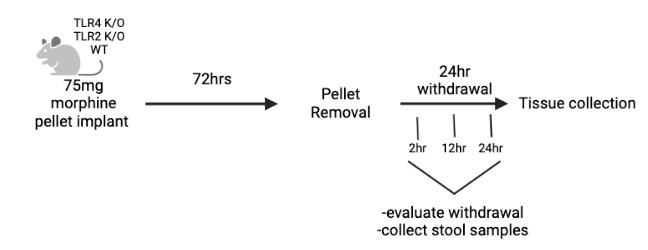
D

E F


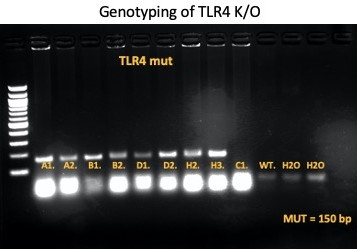

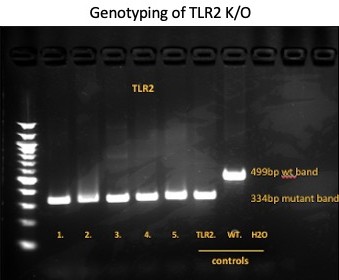

Supplement: Supplemental Material [file KGMI_A_2242610_SM9734.docx]
