# Supplementary material for: The gut microbiome contributes to somatic morphine withdrawal behavior and implicates a TLR2 mediated mechanism
Source: Gut Microbes. 2023 Aug 17;15(1):2242610. doi: 10.1080/19490976.2023.2242610 (PMC10438851; doi:10.1080/19490976.2023.2242610)
Supplement: Supplemental Material [file KGMI_A_2242610_SM9733.docx]

**Supplemental Figures Legends:**

**Supplemental Figure 1**: Dependence drug treatment paradigm with two groups: morphine (MORPH) and placebo (PLCB) (A). Mean of individual symptoms of withdrawal, jumping (B), shaking (C), and grooming (D) are plotted. N=5 per group, no replicates. Statistical analysis by students t-test, *p<0.05, **p<0.01. Box plots of the phylum relative abundance analyzed with T-test and Mann-Whitney test (E). No differences shown are significant and p values are shown above graph. There is an n=7-10 per group. Replicates were done with an n=2-3 per group repeated 4 times. Density representation of phylum in placebo treated mice (F).

**Supplemental Figure 2**: Schematics of antibiotic drug treatment for 4 groups: antibiotic and morphine (ABX+MORPH), antibiotic and placebo (ABX+PLCB), water and morphine (H2O+MORPH) and water and placebo (H2O+PLCB) (A&E). Individual symptoms of withdrawal in antibiotic treated animals. . Means of individual symptoms of withdrawal, shaking (B&G), grooming (C&H), and jumping (F). for each antibiotic treatment means are plotted with SEM error bars. Statistical analysis by 2way ANOVA, Tukey’s multiple comparisons test, *p<0.05, **p<0.01 compared between the antibiotic+morphine and water+morphine treated groups. N=9-11 per group with 4 replicates of 2-3per group. Mass spectrometry results of circulating morphine metabolite M-3-Glu (D), n=4-5 per group.

**Supplemental Figure 3**: Validation of knockout models. Germ-free mouse drug paradigm (A). Means of individual symptoms of morphine withdrawal, shaking (B) and grooming (C) in germ-free animals plotted with SEM error bars. Statistical analysis by 2way ANOVA, Tukey’s multiple comparisons test. There were no significant differences between the GF and SPF groups at any timepoint. There was an n=6 per group. TLR4 and TLR2 whole body knockout model paradigm (D). Genotyping results of the knockout models (E&F).
